# Reassessing Cisplatin Eligibility in Urothelial Carcinoma: A Retrospective Study on Dose Reduction Strategies

**DOI:** 10.7759/cureus.82261

**Published:** 2025-04-14

**Authors:** Satoshi Yamamoto, Koichiro Kurokawa, Koki Watanabe, Sanji Kanaoka, Kazuyoshi Nakamura

**Affiliations:** 1 Urology, Kimitsu Chuo Hospital, Kisarazu, JPN

**Keywords:** cisplatin, dose reduction, renal impairment, treatment efficacy, urothelial carcinoma

## Abstract

Background

This study aims to assess the impact of reduced cisplatin doses on treatment efficacy and toxicity in patients with urothelial carcinoma (UC) and renal impairment. Current guidelines suggest excluding patients with renal impairment from cisplatin treatment. However, reevaluating the efficacy of dose-adjusted cisplatin based on renal function is crucial.

Methods

A retrospective study was conducted on 68 patients diagnosed with locally advanced or metastatic UC (T2-4N0M0, TanyN1-3M0 or TanyNanyM1) between 2017 and 2024. These patients were divided into two groups: 40 received a full dose (100%) of cisplatin (CDDP:70-135mg) and 28 received a 75% dose (CDDP:60-120mg). Clinical outcomes and adverse effects were analyzed and compared between these two groups, with a particular focus on patients with renal impairment (estimated glomerular filtration rate (eGFR) 50-60).

Results

Complete response (CR) rates were 15.0% for the full-dose group and 14.3% for the 75% dose group, while partial response (PR) rates were 35.0% and 42.9%, respectively. The overall response rates (CR + PR) were 50.0% for the full dose and 57.2% for the 75% dose. For patients with renal impairment, the 75% dose group demonstrated similar efficacy and adverse effect profiles compared to the full-dose group.

Conclusions

The findings suggest that reduced doses of cisplatin, based on renal function, do not compromise treatment efficacy in UC patients. Thus, expanding cisplatin eligibility to include UC patients with renal impairment (eGFR 30-60 mL/min) is warranted, indicating that dose-adjusted cisplatin treatment is a viable option.

## Introduction

Platinum-based chemotherapy has traditionally been the cornerstone of treatment for urothelial carcinoma (UC). However, recent findings from the EV302 trial [1] and the CheckMate 901 trial [2] suggest a significant shift in the first-line treatment of metastatic UC.

Currently, there are no criteria for determining ineligibility for the enfortumab vedotin (EV) plus pembrolizumab (Pem) regimen. In ineligibility cases, platinum-based chemotherapy should be considered. It has been reported that approximately 30-50% of UC patients are ineligible for cisplatin administration [3-5].

For patients with renal impairment, one guideline for dose reduction of cisplatin (CDDP) is provided by Kintzel et al. [6]. Their study reported that patients with a creatinine clearance (CCr) of ≥60 mL/min received the full cisplatin dose, while those with CCr of 46-60 mL/min and 30-45 mL/min received 75% and 50% of the full dose, respectively. In 2022, the Japanese Society of Nephrology (JSN) similarly recommended a 75% dose reduction for patients with a CCr of 30-49 mL/min [7].

To mitigate nephrotoxicity, hydration and adequate urine output should be ensured. If necessary, diuretics such as mannitol or furosemide should be administered. Additionally, magnesium supplementation can help prevent cisplatin-induced hypomagnesemia and nephrotoxicity.

However, according to the current Urological Society guidelines [8-10], patients with renal impairment (GFR <60 mL/min) are deemed ineligible for cisplatin. This is primarily due to the lack of clinical outcome analyses evaluating the impact of cisplatin dose reduction in UC patients with moderate renal dysfunction (CCr 30-60 mL/min).

In the neoadjuvant setting, guidelines [8-10] strongly recommend cisplatin-based chemotherapy, while carboplatin is not recommended. Furthermore, Galsky et al. [11] reported that, in vitro, cisplatin exerts a stronger antitumor effect than carboplatin by directly modulating immune responses, enhancing dendritic cell activation, and promoting antigen-specific T-cell cytotoxicity. These findings further support the preferential use of cisplatin.

The CheckMate 901 trial was limited to cisplatin-eligible cases, which significantly restricted the patient population [2]. If this study demonstrates the potential for expanding cisplatin eligibility, it may allow for its application in patients previously deemed ineligible in the CheckMate 901 trial [2]. Moreover, data from the EV-302 trial revealed that cisplatin-ineligible cases exhibit shorter overall survival (OS) compared to cisplatin-eligible cases (cisplatin-eligible: 36.7 months; cisplatin-ineligible: 25.6 months), underscoring the need for additional treatment options for such cases [1]. Furthermore, findings from the JAVEIN Bladder100 trial demonstrated that cisplatin achieves superior outcomes compared to carboplatin when used with avelumab [12]. These considerations collectively highlight the importance of reassessing cisplatin eligibility, which holds significant promise for advancing the treatment landscape of UC.

Given these considerations, is it appropriate to categorically classify all UC patients with renal impairment as ineligible for cisplatin? To address this question, we investigated whether dose reduction of cisplatin in the GC regimen (gemcitabine (1000mg/m²) and cisplatin (70mg/m²), based on GFR values, affects treatment efficacy and toxicity compared to full-dose administration in UC patients.

## Materials and methods

Between 2017 and 2024, we retrospectively identified 68 patients with locally advanced or metastatic UC (T2-4N0M0, TanyN1-3M0 or TanyNanyM1) who underwent GC therapy (gemcitabine (1000mg/m²) and cisplatin (70mg/m²) at Kimitsu Chuo Hospital, including those who received neoadjuvant chemotherapy. Diagnosis was confirmed by primary tumor resection or biopsy, cytology, or metastatic lesion biopsy. Patients who received a 50% dose reduction of cisplatin (CDDP 35mg/m^2^), those who underwent adjuvant chemotherapy, and those treated with carboplatin were excluded. To clarify, the estimated glomerular filtration rate (eGFR) was calculated based on the blood test conducted on the day prior to administration. In patients with an e-GFR of 50 ≦ e-GFR < 60, there has been ongoing discussion regarding the appropriate dosing protocol, specifically whether to administer 75% (CDDP: 52.5mg/m²) or the full dose (CDDP: 70mg/m²). Until 2022, treatment for patients within this e-GFR range involved a 75% dose. Since 2022, following the recommendations issued by the JSN, the full dose has been adopted as the standard protocol for this group.

Among the included patients, 40 received an initial full dose (100%) of cisplatin (N=40), while 28 received a 75% dose (N=28). Most cases were assigned accordingly. We analyzed the clinical background, therapeutic efficacy, and adverse effects of GC chemotherapy in both groups.

Additionally, we evaluated treatment efficacy and adverse effects in patients with impaired renal function (50 ≦e-GFR < 60) by comparing those who received a full 100% dose of CDDP with those who received a 75% dose. The eGFR was calculated using the formula recommended by JSN7. Since CCr may diverge from GFR in patients with renal dysfunction, the eGFR was used as per JSN recommendations.

The GC regimen used in this study was as follows: For UC patients with normal renal function, gemcitabine (1,000 mg/m²) was administered on days 1 and 8, and cisplatin (70 mg/m²) on day 2, every 21 or 28 days. Based on a modified application of Kintzel’s criteria, patients with an eGFR ≥50 mL/min received either the full or a reduced dose of cisplatin (CDDP: 52.5mg/m²), while those with an eGFR of 30-49 mL/min received 50% or 75% of the standard dose. In contrast, gemcitabine dosage was not affected by eGFR levels.

If grade 3 or 4 adverse events occurred repeatedly and a decline in the patient’s general condition was anticipated due to side effects, the dose was reduced to approximately 75% at the physician’s discretion. Granulocyte colony-stimulating factor was considered when the neutrophil count fell below 500/mm³ but was not used prophylactically. The GC dose for each patient was calculated as the average dose across all treatment cycles.

Response evaluation

The tumor size was radiologically assessed using CT scans or MRI prior to initiating the GC regimen. All patients underwent response evaluation after completing several treatment cycles. Tumor response was assessed based on the Response Evaluation Criteria in Solid Tumors (RECIST) version 1.1. Complete response (CR) was defined as the complete disappearance of all signs of cancer for at least four weeks. Partial response (PR) was defined as a ≥30% reduction in the sum of the longest diameters of target lesions without the emergence of new lesions. Stable disease (SD) was defined as a reduction of <30% or an increase of <20% in the sum of the longest diameters of target lesions, without the appearance of new lesions. Progressive disease (PD) was defined as a ≥20% increase in the sum of measurable lesions or the appearance of new lesions. Toxicity was assessed according to the Common Terminology Criteria for Adverse Events (CTCAE) version 5.0. Any unfavorable and unintended sign (including abnormal laboratory findings), symptom, or disease observed during treatment or procedures, regardless of causality. This includes events deemed to have a causal relationship as well as those judged not to have one. The best treatment response was determined based on chemotherapy efficacy through the fourth course. Patients who received neoadjuvant chemotherapy were evaluated using pathological specimens.

Statistical analysis

Radical surgeries included total cystectomy for bladder cancer and nephroureterectomy for upper tract UC. Local recurrence was defined as retroperitoneal recurrence of bladder cancer or upper tract UC. Distant metastases were classified into liver, lung, bone (including bone marrow), and other organs (including adrenal glands, peritoneum, skin, and other unspecified organs). Lymph node metastasis was defined as the presence of regional or distant lymphadenopathy.

Clinical and pathological parameters were evaluated using the chi-square test and Student’s t-test. The distribution of binary and non-ordered categorical variables was compared using the chi-square test, with significance set at P < 0.05. Statistical analyses were performed using the JMP version 18.0 software package (JMP Statistical Discovery LLC, Cary, USA).

## Results

Patient characteristics and eGFR by the CDDP dose administered 

Patient characteristics are summarized in Table 1. The median observation period was 20.2 months (range: 3.1-83.9 months). Among the 68 patients, 40 received the full 100% dose, while 28 received 75% of the dose. Of those who received the 100% dose, nine patients had their dose reduced to 75% due to adverse effects. In the 75% dose group, no further dose reductions were made, but the dosing interval was extended. The median eGFR and the distribution for each group are shown in Table 1.

**Table 1:** Clinical features according to the CDDP dose administered (CDDP 100%; 70mg/m2, CDDP 75%; 52.5mg/m2) Statistically significant: *χ2 test, **Student’s t-test CDDP: cisplatin; PS: performance status; UTUC: upper tract urothelial carcinoma; UC: urothelial carcinoma; AKI: acute kidney injury

Characteristics		CDDP 100%	CDDP 75%	p-value	χ^2^-value	t-value
		n=40(%)	n=28(%)			
Sex	Male	33(82.5)	22(78.6)			
	Female	7(17.5)	6(21.4)	0.68	0.163	
Age	65<	34(85.0)	25(89.3)			
	65≧	6(15.0)	3(10.7)	0.61*	0.263	
PS	0	36(90.0)	23(82.1)			
	1	4(10.0)	5(17.9)	0.35*	0.871	
eGFR	Median(range)	68(50-110)	49(33-57)	<0.001**		-7.67
	eGFR≧60	27(67.5%)	0(0.0%)	<0.001*	40.91	
	50≦eGFR<60	13(32.5%)	16(57.1%)	0.38*	0.757	
	30	0(0.0%)	12(42.9%)	<0.001*	35.95	
Primary site	UTUC	8(20.0)	13(46.4)			
	bladder	29(72.5)	13(46.4)			
	Both	3(7.5)	2(7.2)	0.09*	15.02	
History of radical surgery	Yes	23(57.5)	10(35.7)			
	No	17(42.5)	18(64.3)	0.08*	3.16	
Pure UC	Yes	34(85.0)	25(89.3)	0.25*	8.95	
Grade	High grade	34(85.0)	19(67.8)			
	unknown	6(15.0)	9(32.1)	0.11*	2.78	
Lymph node metastasis	Yes	14(35.0)	13(46.4)			
	No	26(65.0)	15(53.6)	0.34*	0.89	
Visceral metastasis	Yes	13(32.5)	11(39.3)			
	No	27(67.5)	17(60.7)	0.57*	0.33	
Local recurrence	Yes	1(2.5)	1(3.5)			
	No	39(97.5)	27(96.5)	0.43*	0.61	
Stage	Bladder Ⅱ	6(13.0)	4(14.3)			
	Bladder Ⅲa	9(22.5)	3(10.7)			
	Bladder Ⅳ	14(35.0)	5(17.9)			
	UTUC Ⅳ	7(17.5)	12(42.8)	0.12*	7.18	
Timing of chemotherapy	Neoadjuvant	14(35.0)	7(25.0)			
	Metastasis	28(65.0)	22(75.0)	0.42*	10.1	
AKI risk classifications	Moderate	10(25.0)	-	-		
	Moderate～high	8(20.0)	-	-		
	High	22(55.0)	-	-		

Treatment efficacy, response rates, and adverse effects by the CDDP dose administered

**Figure 1:**
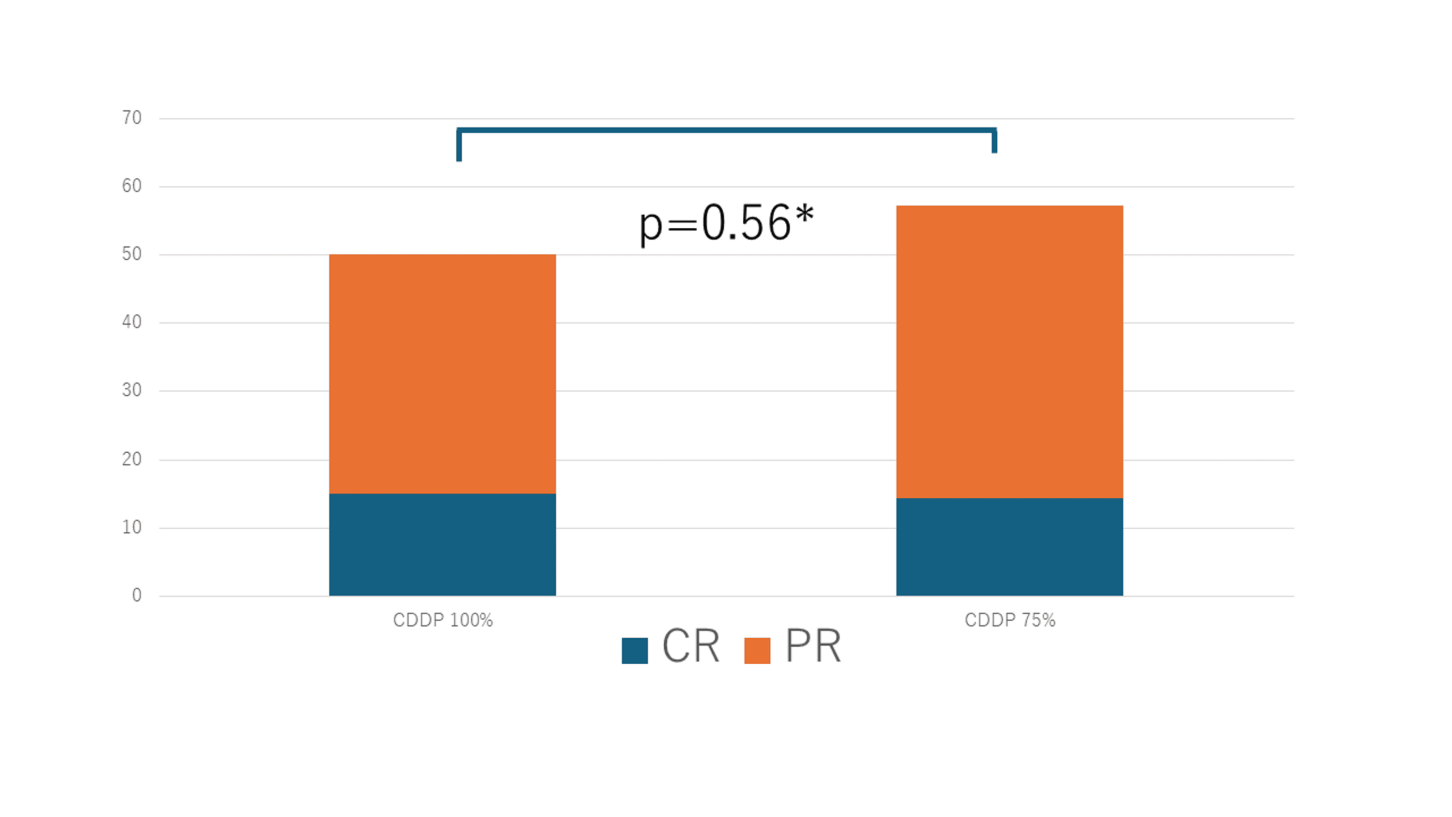
Response rate according to the CDDP dose administered Overall response rate of the two regimens (100% dose GC and 75% dose GC). ^*^The significance test used to compare the two groups was the χ2 test. CR: Complete response; GC: gemcitabine/cisplatin; PR: partial response

The CR rates for the 100% and 75% CDDP doses were 15.0% and 14.3 %, respectively, while the PR rates were 35.0% and 42.9%, respectively (Table 2). The overall response rate (CR + PR) was 50.0% for the 100% dose and 57.9% for the 75% dose (Figure 1).

**Table 2:** Clinical responses according to the CDDP dose administered CDDP: Cisplatin; CR: complete response; PR: partial response; SD: stable disease; PD: progressive disease

Responses		CDDP 100%	CDDP 75%
		n=40(%)	n=28(%)
CR		6(15.0)	4(14.3)
PR		14(35.0)	12(42.9)
SD		6(15.0)	2(7.1)
PD		14(35.0)	10(35.7)

Furthermore, treatment efficacy was compared between the 100% and 75% CDDP doses for patients with an eGFR between 50 and 60. Patient characteristics for this subgroup are provided in Table 3.

**Table 3:** Clinical features according to the CDDP dose administered (50≦eGFR<60) Statistically significant: *χ2 test CDDP: cisplatin; PS: performance status; UTUC: upper tract urothelial carcinoma; UC: urothelial carcinoma

Characteristics 50≦eGFR<60		CDDP 100%	CDDP 75%		χ^2^-value
		n=13(%)	n=12(%)	p-value	
Sex	Male	10(76.9)	8(66.7)		
	Female	3(23.1)	4(33.3)	0.57*	0.326
Age	65<	11(84.6)	10(83.3)		
	65≧	2(15.4)	2(16.7)	0.93*	0.008
PS	0	10(76.9)	10(83.3)		
	1	3(23.1)	2(16.7)	0.69*	0.161
Primary site	UTUC	4(30.8)	5(41.7)		
	Bladder	8(61.5)	6(50.0)		
	Both	1(7.7)	1(8.3)	0.83*	0.358
History of radical surgery	Yes	7(53.8)	3(25.0)		
	No	6(46.2)	9(75.0)	0.13*	2.21
Pure UC	Yes	12(92.3)	10(83.3)	0.36*	3.14
Grade	High grade	10(76.9)	8(66.7)		
	Unknown	3(23.1)	4(33.3)	0.56*	0.326
Lymph node metastasis	Yes	5(38.5)	6(50.0)		
	No	8(61.5)	6(50.0)	0.56*	0.338
Visceral metastasis	Yes	4(30.8)	4(33.3)		
	No	9(69.2)	8(66.7)	0.89*	0.019
Local recurrence	Yes	0(0.0)	1(8.3)		
	No	13(100.0)	11(91.7)	0.21*	1.51
Stage	bladder Ⅱ	2(15.4)	2(16.7)		
	bladder Ⅲa	1(7.69)	0(0.0)		
	bladder Ⅳ	5(38.5)	4(33.3)		
	UTUC Ⅳ	3(23.1)	4(33.3)	0.81*	1.60
TIming of chemotherapy	Neoadjuvant	3(23.1)	2(16.7)		
	metastasis	10(76.9)	10(83.3)	0.37*	6.45

**Figure 2:**
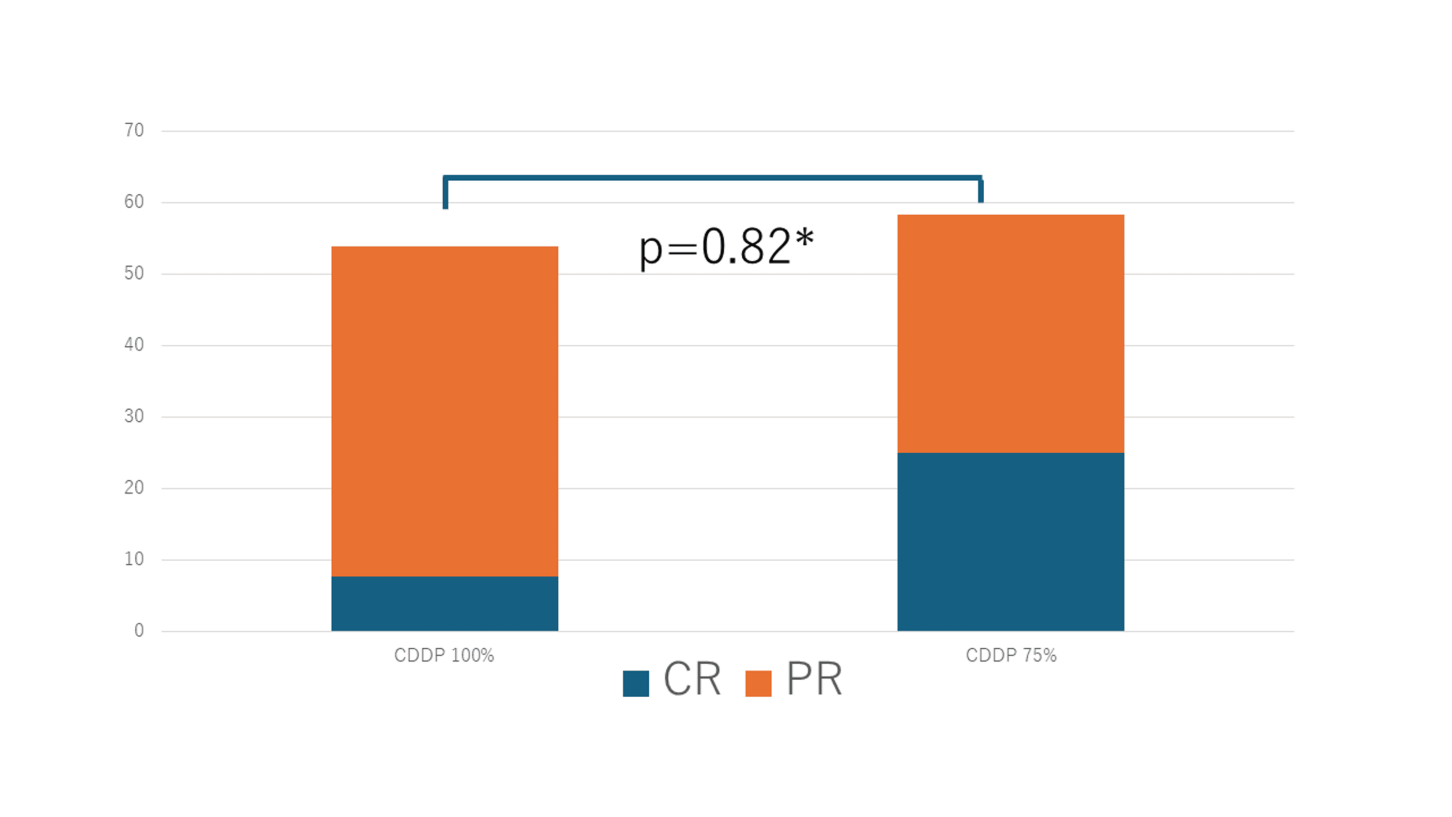
Response rate according to the CDDP dose administered (50≦eGFR<60) Overall response rate of the two regimens (100%-dose GC and 75% dose GC) for patients with an eGFR between 50 and 60. ^*^The significance test used to compare the two groups was the χ2 test. CR: complete response; GC: gemcitabine/cisplatin; PR: partial response

The CR rates for the 100% and 75% doses were 7.7% and 25.0%, respectively, while the PR rates were 46.1% and 33.3%, respectively (Table 4). The overall response rate (CR + PR) was 53.8% for the 100% dose and 58.3% for the 75% dose (Figure 2).

**Table 4:** Clinical responses according to the CDDP dose administered (50≦eGFR<60) CDDP: cisplatin; CR: complete response; PR: partial response; SD: stable disease; PD: progressive disease

Responses 50≦eGFR<60		CDDP 100%	CDDP 75%
		n=13(%)	n=12(%)
CR		1(7.7)	3(25.0)
PR		6(46.1)	4(33.3)
SD		2(15.4)	1(8.3)
PD		4(30.8)	4(33.3)

Regarding adverse effects, grade 3 or higher toxicities are detailed in Table 5. Acute kidney injury was observed in one patient each in the 100% and 75% dose groups. In the 100% dose group, the risk classification for acute kidney injury included 22 patients (55.0%) in the high-risk category, 8 patients (20.0%) in the medium-to-high-risk category, and 10 patients (25.0%) in the medium-risk category. Notably, all patients who experienced acute kidney injury were in the high-risk category. The acute kidney injury is detailed in Table 6. Since risk classification for acute kidney injury was not applicable to patients with an eGFR <50, risk stratification was not performed in the 75% dose group.

**Table 5:** Adverse effect profiles according to the CDDP dose administered CDDP: cisplatin

		CDDP 100%	CDDP 75%
		n=40(%)	n=28(%)
	Toxicity	Grade3/4	Grade3/4
Leukopenia		12(30.0)	9(32.1)
Neutropenia		11(27.5)	10(35.7)
Thrombocytopenia		12(30.0)	9(32.1)
Hyponatremia		2(5.0)	2(7.1)
Hypokalemia		1(2.5)	0(0.0)
Febrile neutropenia		1(2.5)	0(0.0)
Acute kidney injury		1(2.4)	1(3.6)

**Table 6:** Acute kidney injury according to the CDDP dose administered Abbreviations：CDDP；cisplatin

Acute kidney injury		CDDP 100%	CDDP 75%
		n=40(%)	n=28(%)
Grade0		29(72.5)	16(57.1)
Grade1		9(22.5)	11(39.3)
Grade2		1(2.5)	0(0.0)
Grade3		1(2.5)	1(3.6)
Grade4		0(0.0)	0(0.0)
Grade5		0(0.0)	0(0.0)

As an additional analysis, Table 7 presents a comparison of the toxicity profiles between full-dose cisplatin and 75% dose cisplatin in patients with an eGFR of 50-60 mL/min/1.73 m².

**Table 7:** Adverse effect profiles according to the CDDP dose administered (50≦eGFR<60) CDDP: cisplatin

50≦eGFR<60		CDDP 100%	CDDP 75%
		n=13(%)	n=12(%)
	Toxicity	Grade3/4	Grade3/4
Leukopenia		4(30.8)	4(33.3)
Neutropenia		4(30.8)	5(41.7)
Thrombocytopenia		4(30.8)	5(41.7)
Hyponatremia		0(0.0)	2(16.6)
Hypokalemia		2(15.4)	0(0.0)
Febrile neutropenia		0(0.0)	0(0.0)
Acute kidney injury		0(0.0)	0(0.0)

## Discussion

In this study, we investigated the oncological outcomes of cisplatin dose reduction due to renal dysfunction in UC patients. To better assess the clinical effects of cisplatin dose reduction, we focused not only on metastatic UC patients receiving first-line GC therapy but also on those undergoing neoadjuvant chemotherapy. We divided patients with UC who received GC therapy into two groups: one receiving 100% cisplatin dose (N=40) and the other receiving 75% cisplatin dose (N=28).

Our UC cohort demonstrated that cisplatin dose reduction did not adversely affect cancer-specific prognosis in UC patients with renal dysfunction, characterized by a GFR of 30-50 mL/min. Moreover, no patients discontinued treatment due to grade 3/4 toxicities, including leukopenia, neutropenia, thrombocytopenia, febrile neutropenia, or acute kidney injury. These results support recommendations from nephrology societies regarding cisplatin dose reduction [6,7,13]. Specifically, dose reduction of gemcitabine plus cisplatin may be a feasible treatment option for metastatic UC patients with an eGFR of 30-50 mL/min. Based on a risk classification using Gupta et al. [14] report on cisplatin-induced nephrotoxicity, 33 patients were classified as high-risk, suggesting that cisplatin dose reduction should be considered for some of these individuals. The incidence of cisplatin-associated acute kidney injury was 7.5% in the high-risk group and 2.7% in the moderate-risk group.

For the moderate-risk group, avoiding nephrotoxic drugs and ensuring adequate hydration were recommended as preventive measures. In the high-risk group, in addition to these strategies, frequent blood testing, dose reduction, and consideration of alternative treatments were advised. However, in our analysis, some high-risk cases lacked magnesium (Mg) test results, leading us to use the term “moderate to high risk” instead.

Indeed, a nationwide survey across 627 Japanese institutions revealed that 54.9% of facilities employed gemcitabine plus cisplatin dose reduction for metastatic UTUC patients with renal dysfunction (GFR 30-59 mL/min), while 27.1% used the GCarbo regimen [15]. However, there is currently no clear, universally accepted protocol for dose reduction.

The treatment landscape for metastatic UC has expanded in recent years [16], though a clearly defined optimal patient profile has not yet been established. According to a report by Barthélémy et al. [17], real-world data indicate that sequential therapy starting with GC followed by atezolizumab and enfortumab vedotin resulted in an overall survival (OS) of 40.8 months, suggesting high treatment efficacy. Moreover, the JAVELIN Bladder 100 Trial, after ≥2 years of follow-up [12], showed that cisplatin-based regimens were more effective than carboplatin-based regimens in first-line treatment. This highlights the continued importance of cisplatin when using immune checkpoint inhibitors for maintenance therapy.

A study using patient-derived biosamples from the IMvigor130 trial [11] demonstrated that cisplatin, compared to carboplatin, upregulated gene expression related to antigen presentation and T-cell activation in immune cells, especially monocytes. Cisplatin also induced stronger expression of immune-related and inflammation-related genes in tumor cells, potentially through the ATR DNA damage transducer. Tumor cells treated with cisplatin showed greater activation of dendritic cells, T-cell proliferation, and enhanced antigen-specific tumor cell killing than those treated with carboplatin. Although both cisplatin and carboplatin form DNA adducts, cisplatin does so more rapidly, resulting in more potent anti-tumor activity. These differences suggest that cisplatin has a stronger immunomodulatory effect, particularly in scenarios where pre-existing adaptive immunity is present, possibly leading to improved clinical outcomes.

However, research on cisplatin dose reduction and split dosing remains limited, and these practices are still under debate. A study on GC dose reduction in metastatic UC patients with renal dysfunction (GFR <60 mL/min/1.73 m²) reported a one-year overall survival (OS) of 26.3% in 25 patients receiving dose reduction, significantly lower than the 60.3% OS in 32 patients receiving the standard dose [18]. However, this study did not clarify the protocol for dose reduction. Maru et al. [19] also reported on the impact of baseline renal function and dose reduction of nephrotoxic chemotherapeutic agents on the outcomes of metastatic UC patients. In their study, the median OS for 22 patients who underwent dose reduction was 10 months, significantly shorter than the median OS of 17 months in 35 patients who received the standard dose. This retrospective study included only three cases from the dose-reduced MVAC and GC regimens.

Izumi et al. [20] analyzed UC patients with renal dysfunction (eGFR 40-60 mL/min) who received either GC split dosing or GCarbo and found that the median OS for the GC split group was 18.1 months, while for the GCarbo group, it was 12.5 months (p=0.0454). The median progression-free survival (PFS) was 9.9 months for GCsplit and 6.4 months for GCarbo (p=0.0404), suggesting that GCsplit could be a promising treatment option for advanced UC patients who cannot receive cisplatin due to renal dysfunction. A network meta-analysis on cisplatin split dosing [21] reported an overall response rate of 39-80%, which was significantly higher than GCa (OR 1.97, 95% CrI 1.29-3.02), with treatment discontinuation rates due to adverse events ranging from 5% to 38%.

Sugimoto et al. [22] reported oncological and toxicological outcomes for full-dose GC, dose-reduced GC, and GCa. Their analysis revealed that, unlike findings in clinical trial settings, there were minimal significant differences in survival and therapeutic efficacy across the three treatment modalities. Possible explanations for these contrasting outcomes include the variability in cisplatin dose reduction and the physician-driven discretion in treatment selection, which may have introduced bias, as treatments were likely tailored to patient-specific characteristics.

However, no studies have compared cisplatin split dosing with cisplatin dose reduction, and further research is needed.

In this study, no significant differences in response rates or toxicity were observed between full-dose and 75% dose cisplatin in patients with an eGFR of 50-60 mL/min/1.73 m². This result is likely influenced by the small sample size. Moreover, previous studies, such as that by Culine et al. [23], have reported a correlation between the total dose of cisplatin and pathologic complete response. Based on these findings, I personally believe that, whenever feasible, full-dose cisplatin administration should be prioritized.

Limitations

This study has several limitations. It was retrospective and non-randomized, with a small sample size, indicating that larger-scale validation is required. The inclusion of only UC patients from a single institution may have introduced selection bias. Especially, the higher response rate observed in the 75% dose group in this study may be influenced by selection bias. Additionally, the response evaluations conducted by multiple radiologists at a single institution could be considered a limitation. Moreover, in metastatic cases, evaluations are performed using imaging studies, while for neoadjuvant cases, postoperative pathological assessments are utilized. However, inconsistencies between these evaluation methods highlight the need for further investigation. Furthermore, we will include the following points in the limitations section. This study exclusively involved Japanese patients, and we were unable to assess the potential impact of racial differences. Also, in the full-dose group, the proportion of high-grade cases was slightly higher, and its potential impact on the results remains unclear. However, considering that the study targets advanced UC, it is probable that most cases involved high-grade tumors. The treatment backgrounds for OS varied considerably due to differences in the eras when pembrolizumab was available as subsequent therapy, when avelumab maintenance therapy was available, and when neither was accessible. As a result, comparing OS was not feasible in this study. Similarly, for PFS, GC therapy was typically limited to 4-6 cycles when avelumab was available. However, in cases where avelumab was not an option, PFS comparisons were not possible.

## Conclusions

Our findings demonstrate that cisplatin dose reduction (75% CDDP: 52.5mg/m²), tailored to renal function, does not negatively impact the clinical outcomes of UC patients undergoing chemotherapy. This result underscores the feasibility of safely administering adjusted doses of cisplatin in patients with renal insufficiency. Furthermore, these findings suggest a reevaluation of cisplatin eligibility criteria, proposing an expansion to include UC patients with moderate renal impairment (eGFR 30-60 mL/min). Such patients, who are conventionally excluded from cisplatin-based regimens due to concerns over nephrotoxicity, may benefit significantly from dose-reduced therapies without compromising therapeutic effectiveness. These insights hold promise for advancing inclusive, personalized treatment strategies that cater to the needs of a broader range of UC patients.
